# Proteome analysis of human gastric cardia adenocarcinoma by laser capture microdissection

**DOI:** 10.1186/1471-2407-7-191

**Published:** 2007-10-11

**Authors:** Yan Cheng, Jun Zhang, Yang Li, Yan Wang, Jun Gong

**Affiliations:** 1 Department of Gastroenterology, the Second Affiliated Hospital, Xi'an Jiaotong University, Xi'an, China; 2 Department of Otolaryngology, Shaanxi Provincial People's Hospital, Xi'an, China

## Abstract

**Background:**

The incidence of gastric cardiac adenocarcinoma (GCA) has been increasing in the past two decades in China, but the molecular changes relating to carcinogenesis have not been well characterised.

**Methods:**

In this study, we used a comparative proteomic approach to analyse the malignant and nonmalignant gastric cardia epithelial cells isolated by navigated laser capture microdissection (LCM) from paired surgical specimens of human GCA.

**Results:**

Twenty-seven spots corresponding to 23 proteins were consistently differentially regulated. Fifteen proteins were shown to be up-regulated, while eight proteins were shown to be down-regulated in malignant cells compared with nonmalignant columnar epithelial cells. The identified proteins appeared to be involved in metabolism, chaperone, antioxidation, signal transduction, apoptosis, cell proliferation, and differentiation. In addition, expressions of HSP27, 60, and Prx-2 in GCA specimens were further confirmed by immunohistochemical and western blot analyses.

**Conclusion:**

These data indicate that the combination of navigated LCM with 2-DE provides an effective strategy for discovering proteins that are differentially expressed in GCA. Such proteins may contribute in elucidating the molecular mechanisms of GCA carcinogenesis. Furthermore, the combination provides potential clinical biomarkers that aid in early detection and provide potential therapeutic targets.

## Background

Various analyses of cancer incidence data culled from Western countries have revealed rapidly rising rates of adenocarcinoma of the esophagus and gastric cardia in the last few decades, compared with the stable and declining rates for esophageal squamous cell carcinoma (SCC) and distal gastric adenocarcinoma (DGA) [1-3]. This phenomenon is also apparent in China, except that the increasing incidence of gastric cardia adenocarcinoma (GCA) appears notably higher than the incidence of esophageal cancer. Evidence indicates that GCA is a distinct clinical entity as its pathogenesis and risk factors are quite different from DGA. Therefore, GCA is far more prevalent, with a higher incidence of lymph node metastasis and a poorer prognosis than DGA [4]. The annual incidence of GCA is 50/100,000 and may even be as high as 190/100,000 in several regions of China [5]. The relatively asymptomatic nature in the early stages of the disease and the lack of adequate screening tests have resulted in a majority of GCA patients diagnosed to be at an already advanced stage of the disease. Thus, it is necessary to understand the molecular mechanism of carcinogenesis and to identify the biomarkers for the early diagnosis and effective treatment of human GCA.

Recently, the proteome has emerged as a complement component of the genome. The supposition is that it could drastically help in unravelling the biochemical and physiological mechanisms of complex multivariate diseases at the functional molecular level. Although genetic mutation and/or errant gene expression may underlie a disease, the biochemical bases for most diseases are caused by protein defects. Therefore, an analysis of global protein abundance in human tumours, called cancer proteomics, could offer many opportunities and challenges in identifying new tumour markers and therapeutic targets as well as in understanding tumour pathogenesis. Currently, two-dimensional gel electrophoresis (2-DE) and mass spectrometry (MS) are the most widely employed tools for separating and identifying proteins. However, heterogeneity is always a concern in studies of human tumour tissue. Although cell culture is one approach to overcome this problem, it might not accurately represent the molecular events taking place in the actual tissue from which they were derived [6]. A comparison between human prostate cell lines and tumour cells from the same patients showed that 20% of the protein profiles were altered [7]. Laser capture microdissection (LCM) is a recent development which can be used to procure highly representative subpopulation of cells from complex heterogeneous tissue samples [8]. This technology has been used very successfully in a diverse array of studies using downstream analysis at the DNA and RNA levels, including global gene expression profiling [9] and analyses of the proteome of prostate [7], colon [10], hepatocellular [11], breast [12], and pancreatic tumours [13]. However, the combination of 2-DE and MS has never been applied to the study of human GCA.

This study aims to outline the carcinogenesis of GCA and to identify GCA-specific disease-associated proteins as potential clinical biomarkers for early detection and new therapeutic targets. We performed navigated LCM to enrich both the malignant and nonmalignant gastric cardiac epithelia cells from paired surgical specimens of human GCA. The proteins extracted from these cells were separated by 2-DE. Differential protein spots were identified by peptide mass fingerprint (PMF) based on matrix-assisted laser desorption/ionisation time-of-flight mass spectrometry (MALDI-TOF MS) and database searching. The validity of these findings was confirmed by immunohistochemical and western-blot analyses.

## Methods

### Materials

IPG strips (pH 3–10 nonlinear) and IPG buffer solutions (pH 3–10 nonlinear) were purchased from Amersham Pharmacia Biotech (APB, Sweden). DTT, urea, thiourea, Tris base, TX-100, CHAPS, glycine, acrylamide, methylenebisacrylamide, SDS, TEMED, ammonium persulfate, silver nitrate, Trypsin (sequencing grade), ACN, and TFA were purchased from Sigma (St. Louis, MO, USA). Finally, the complete protease inhibitor cocktail was from Roche (Lewes, UK). Milli-Q grade water was used for all the solutions.

### GCA samples

Nine pairs of human gastric cardia adenocarcinoma and their adjacent nontumourous cardia tissues were obtained within 30 minutes after a surgical resection at the second affiliated hospital of Xi'an Jiaotong University in 2005 (Table 1). In order to avoid obvious areas of ulcer or necrosis, the samples were procured from the edges of the tumours. They were immediately frozen in liquid nitrogen and stored at -80°C until use. Informed consents were obtained from all patients. Meanwhile, two experienced pathologists evaluated the tumour grading by a microscopic examination of the samples.

**Table 1:** Clinical and histological data of patient tumour samples

Case	Age	Gender	Location^a)^	Size(cm)	Grade
1	58	M	Within 2 cm of GEJ	1.5 × 2	Moderately differentiated
2	63	M	Within 2 cm of GEJ	5 × 6	Moderately differentiated
3	46	M	Within 2 cm of GEJ	4 × 5	Moderately differentiated
4	60	M	Within 2 cm of GEJ	2 × 2.5	Well differentiated
5	73	M	Within 2 cm of GEJ	3 × 1	Well differentiated
6	70	M	Within 2 cm of GEJ	2 × 1	Well differentiated
7	55	M	Within 2 cm of GEJ	2 × 3	Poorly differentiated
8	51	M	Within 2 cm of GEJ	4 × 6	Poorly differentiated
9	63	M	Within 2 cm of GEJ	3 × 2.5	Poorly differentiated

a) Location: epicenter of tumour tissue

### Sample preparation for LCM

Sections (8 um thick) were cut onto slides (precleaned using a detergent, washed with deionised water, and dipped in ethanol) using a Leica CM 1900 cryostat (chamber temperature -28°C). The sections were either stored at -80°C or kept in the cryostat chamber prior to LCM. Haematoxylin staining was carried out only for monitoring of tissue sections and was not used in conjunction with LCM. Sections were fixed (70% ethanol for 1 min), haematoxylin stained and dehydrated (70% ethanol for 45 s, 95% ethanol for 45s, 2 × 100% ethanol for 30s, and followed by xylene 2 × for 5 min). Meanwhile, unstained sections were used for LCM. They were fixed (70% ethanol for 30s), washed in deionised water for 10s, and dehydrated (70% ethanol for 15s, 95% ethanol for 15s, 2 × 100% ethanol for 15s, and followed by xylene 2 × for 1 min). Complete protease inhibitor cocktail tablets were

### Navigated LCM

After fixing and dehydration, unstained sections were air dried and microdissected using the Arcturus PixCellα system (Arcturus, Mountain View, CA, USA). LCM microscope imaged a haematoxylin stained section adjacent to the one of interest. This image was displayed on the LCM computer screen using LCM image analysis software (Arcturus, USA) and was used as a map to delimit the region for dissection on an adjacent unstained section. The sections were then captured using a 15 um diameter laser beam typically at 50–80 mV power with pulse duration of 3–10 ms in machine gun mode and with a laser firing frequency of 1 shot per 500 ms. On the average, between 10,000–12,000 shots were taken per cap, and approximately 25,000–30,000 cells were obtained per cap. Each cap was captured within one hour. Based on a careful review of the histologic sections, each dissection was estimated to contain ≥ 95% of the desired cells.

Microdissection caps were inserted into 0.5 mL microcentrifuge tubes containing 50 uL of a lysis buffer (7 M urea, 2 M thiourea, 4% w/v CHAPS, 65 mM DTT, 0.5% v/vTX-100, 40 mMTris-Base, and complete protease inhibitor cocktail). Then the cells were solubilised by inversion of tubes followed by vortex-mixing for 1 min and brief pulse-centrifugation at 12, 000 × g. Afterwards, tissues from multiple caps (about 800,000~1,000,000 cells) were extracted into the same lysis buffer until sufficient material had been collected. The samples were centrifuged at 40,000 × g for 1 hour at 4°C. Where necessary, supernatants were stored at -80°C until further use. Protein concentration was determined by the Bradford method.

### 2-DE

The first-dimensional isoelectric focusing (IEF) was carried out on Pharmacia Immobiline IPG DryStrip system (Uppsala, Sweden). For the first dimension of electrophoresis, the samples containing 60 ug protein for analysis gels were diluted to 250 uL with a rehydration solution (7 M urea, 2% w/v CHAPS, 50 mM DTT, 0.5% v/v IPG buffer (pH 3–10 nonlinear), and trace bromophenol blue) before loading onto 13 cm IPG strips (pH3–10 nonlinear). IEF was then performed using IPGphor electrophoresis unit according to the manufacturer's instructions. Thereafter, the strips were equilibrated with a solution (6 M urea, 30% v/v glycerol, 2% w/v SDS, and 50 mM Tris-HCl, pH 8.8), reduced with 1% w/v DTT for 15 min, and alkylated with 2.5% w/v iodoacetamide for 15 min. Strips were then rinsed in electrophoresis buffer (25 mM Tris base, 192 mM glycine, and 0.1% w/v SDS), applied to 11% acrylamide gels, and sealed with melted agarose (0.5% w/v agarose in electrophoresis buffer containing a trace of bromophenol blue). SDS-PAGE was carried out using Hoefer SE 600 vertical chambers and a Tris-glycine buffer (25 mM Tris and 192 mM glycine) containing 0.1% w/v SDS, with initial separation at a constant 10 mA/gel for 30 min followed by 20 mA/gel. The second-dimensional SDS-PAGE was developed until the bromophenol blue dye marker had reached the bottom of the gel. The total run time was typically 4 to 4.5 hours. Gels were fixed in 10% v/v acetic acid, 40% v/v ethanol before sensitisation for 30 min in a buffer containing 30% v/v ethanol, 0.2% w/v sodium thiosulphate, and 0.83 M sodium acetate. This was followed by three 15 min washes in deionised water. The proteins were then stained with 0.1% w/v silver nitrate for 20 min, washed twice in deionised water for 1 min, and developed in 2.5% w/v sodium carbonate containing 0.04% v/v formaldehyde (37% solution). The development was stopped with 1% v/v acetic acid, and the gels were washed three times in water.

### Image analysis and statistical analysis

Umax ImageScanner (Amersham Biosciences) was used to scan the gels, while Image Master™ 2D Platinum Version 5.0 software (Amersham Biosciences) was used for spot detection, quantification, and matching. The intensity of each spot was quantified by % volume. Data were then analysed using SPSS for Windows 11.5 and Excel. Student's *t*-test was used to analyse the differences in protein levels between GCA and non-tumour samples, with a confidence level of 95%.

### MALDI-TOF-MS and database search

Consistently and significantly different spots selected for analysis by MALDI-TOF MS. Protein spots of interest were excised and minced into small pieces followed by destaining and washing with deionised water for several times until the yellow color disappeared. Gel pieces were then rinsed with 25 mM ammonium bicarbonate, dehydrated with ACN, and dried in a vacuum centrifuge. Afterwards, proteins in-gel were digested with 0.02 ug/uL trypsin overnight at 37°C. Tryptic peptides were then re-dissolved in a solution containing 50% v/v ACN and 0.2%v/v TFA. A 2 uL aliquot was spotted onto a sample plate with 4 uL of matrix solution CHCA (a-cyano-4-hydroxcinnamic acid, 10 mg/mL in 50% v/v ACN, 0.2%v/v TFA) and allowed to air dry. The dried spots were then analysed in a Voyager DE MALDI-TOF MS (Framingham, MA, USA). This was followed by running the spectrometer in a positive ion mode and in reflector mode with the following settings: accelerating voltage, 20 KV; gride voltage, 94%; guide wire voltage, 0.01%; and a delay of 200 ns. Spectra were acquired manually with the laser intensity set at 3200 with 80 shots per spectrum. Then the mass range was set between *m*/*z *500 and *m*/*z *5000. Internal calibration was applied using angiotensin II and insulin B chain peaks at 912.08 Da and 3495.95 Da, respectively.

Proteins were identified by peptide mass fingerprinting using the search program Aldente [14] with the following search parameters applied: SWISS-PROT and TrEMBL were used as the protein sequence databases; a mass tolerance of 100 ppm and one incomplete cleavage were allowed; carboxyamidomethylation, oxidised methionine, and phosphorylation were considered as possible modifications; the minimum number of matched-peptides was 4; and the peptide ion was [M + H]^+^.

### Immunohistochemical and western blot analysis

To validate the expression patterns of three proteins in GCA tissues, immunohistochemistry was performed using formalin-fixed and paraffin-embedded tissue specimens that were matched with 2-DE samples. Dewaxed 5 um thick sections were treated with a 0.3% hydrogen peroxidase for 3 min and with a blocking antibody for 30 min. After heat-mediated antigen retrieval, sections were incubated with primary antibody at 4°C overnight as follows: HSP27 (Sigma, St. Louis, MO, USA), 1:500; HSP60 (Santa Cruz, America), 1:300; and Prx-2 (Santa Cruz, America), 1:150. Sections were then incubated with a peroxidase-labeled antibody (1:500), developed with diaminobenzine, and counterstained with hematoxylin.

For western blot analysis, the protein samples (30 ug) used in 2-DE were run on 12% SDS-PAGE, transferred onto PVDF membranes, and then blocked with PBS/5% skim milk/0.01% Tween for 2–4 hours at room temperature. Primary antibody was diluted in blocking buffer and was added as follows: HSP27, 1:200; HSP60, 1:300; and Prx-2, 1:200. Afterwards, it was incubated with a horseradish peroxidase (HRP)-conjugated secondary antibody and a HRP-conjugated anti-GAPDH/β-actin antibody to confirm equal protein loading in each lane for 1–2 hours at 37°C or room temperature. The samples were washed and detected with enhanced chemiluminescence for 30–60 s (Minipore).

## Results

### Profile differences between navigated LCM samples and whole undissected cryostat tissues

**Figure 1:**
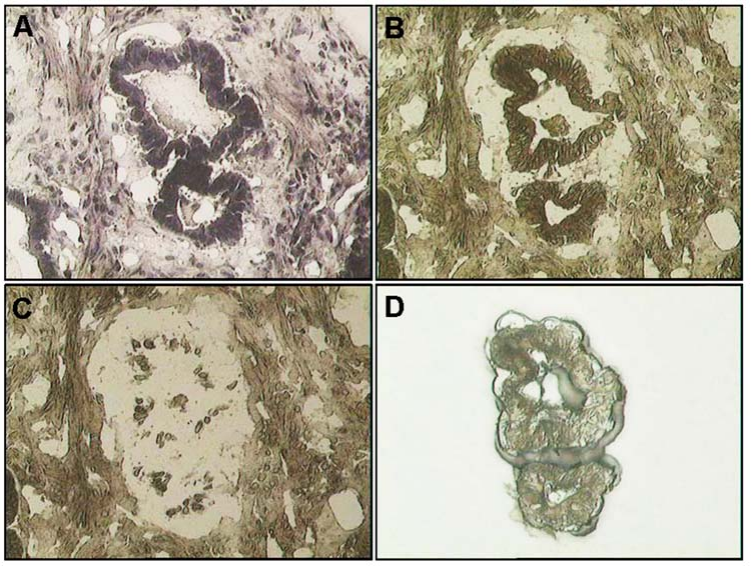
**Navigated laser capture microdissection of tumour tissue**. (A) Haematoxylin-stained gastric cardia adenocarcinoma; (B) Unstained GCA tissue; (C) Tumour tissue after microdissection; (D) Captured tumour cells on LCM film.

To evaluate the effects of navigated LCM on the profiles of tissue protein, we performed 2-DE protein profiles of whole undissected cryostat tissues and LCM-samples of GCA. Figure 1 shows an example of the navigated LCM process. Majority of the spots detected in the dissected non-tumour and tumour tissues could be observed in the whole undissected cryostat tissues, except for several spots. However, there are still some spots found in the whole undissected cryostat tissue which could not be observed in both LCM samples. Thus, this technology could not only enrich non-tumour and tumour epithelial cells but could also diminish the contamination in tissues such as hemoglobin [10,11]. Moreover, the navigated LCM process could eliminate the effects of staining on protein separation by 2-DE [15]. Thus, our data support the need to perform navigated LCM in the proteomic studies of GCA tissues.

### Profile differences in protein expression between GCA tissues and surrounding non-tumour tissues

**Figure 2:**
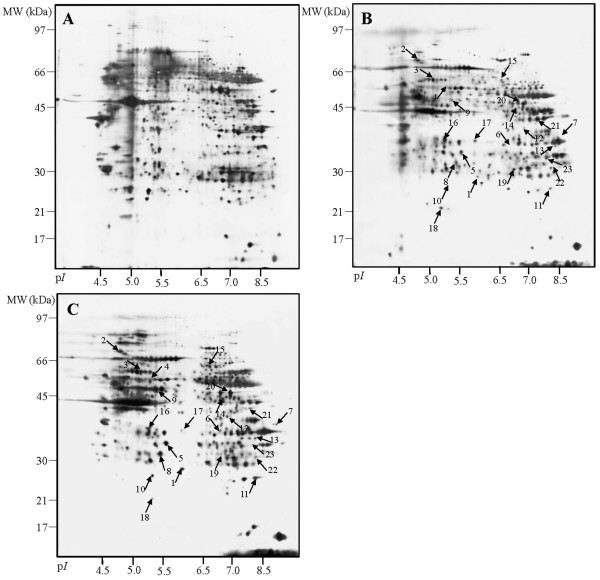
**2-DE map of malignant and nonmalignant gastric cardia tissue**. All the maps shown are prepared from the same patient. (A) 2-DE map of whole undissected cryostat tissues; (B) 2-DE map of non-tumour tissue after LCM; (C) 2-DE map of GCA tissue after LCM.

We performed a navigated LCM and 2-DE for matched pairs of tumour tissues and the surrounding non-tumour tissues from GCA patients. Approximately 800–1,000 protein spots were detected by silver staining. To measure reproducibility, each sample underwent the experiment three times. There were 905 ± 74 and 867 ± 51 protein spots in the map of GCA and non-tumour gastric cardiac tissue, respectively. The matched spots were 799 ± 29 and 727 ± 34 separately, and the respective average matching rate was 88.2% and 83.8%. In addition, the average position deviation of matched spots was 1.031 ± 0.205 mm and 1.44 ± 0.11 mm in the IEF and SDS-PAGE direction, respectively. Although the image analysis showed that these 2-DE maps were reproducible, there were slight differences in the intensity and number of spots. Nonetheless, an analysis of the gels revealed 27 protein spots whose intensities varied substantially and consistently between non-tumour and tumour tissues (Fig. 2). The ratios of normalised spot intensities of cancer to paraneoplasis tissues for each of the proteins of interest were calculated. Spots showing a two-fold difference and with statistical significance (p < 0.05) were selected. Three of the 2-DE results were confirmed by immunohistochemical and western blot analyses.

### Protein identification

**Figure 3:**
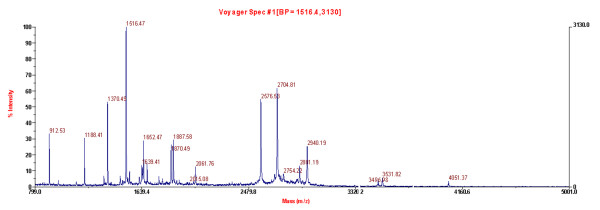
MALDI-TOF MS identification of spot 3 in GCA.

Both whole undissected cryostat specimens and LCM-specimens were used for protein identification. Twenty-seven different protein spots between GCA tissues and surrounding non-tumour tissues were excised. However, only 23 proteins were identified by MALDI-TOF-MS (Table 2). This phenomenon is likely due to post-translational modifications like peroxiredoxin 1 (Prx-1), which was present in two adjacent spots (Fig. 2, spot 11). These proteins were classified into any of the following: cell proliferation and differentiation (ANXA2, ANXA4, hnRNPA2/B1), apoptosis (Prx-1, Prx-2, GSTP, VDAC, ETFB), metabolism (ADH1C, AKR1C3, CA2, GATM), protease related (CTSD, PACSIN1), cystoskeleton (Actin 1, Keratin 8), chaperones (HSP27, 60, 70, PDIA3), and RNA binding and transcription (hnRNPH3, PCBP1, ENO1). Figure 3 shows the PMF maps of HSP60.

**Table 2:** Proteins with differential expression between GCA tumour tissues and adjacent paired non-tumour tissues were identified by MALDI-TOF MS

Spot No.	Protein	Accession No.^a)^	Mr/pI^b)^	Match	Cov (%)^c)^	T-test	Ration (tumour/non-tumour) Means ± SD
**Up-regulation proteins**							
1	Heat-shock protein beta-1(HSP27)*	P04792	23/6.0	9	54	0.0040	3.4279 ± 2.0727
2	Heat shock 70 kDa protein 5(HSP70)	P11021	70/5.0	22	42	0.0030	3.2597 ± 1.5314
3	60 kDa heat shock protein(HSP60)*	P10809	58/5.2	12	37	0.0001	2.8308 ± 1.1426
4	Protein disulfide isomerase A3(PDIA3)*	P30101	54/5.6	13	41	0.0280	4.2105 ± 2.7294
5	Annexin A4(ANXA4)	P09525	36/5.8	10	34	0.0140	4.7153 ± 7.3434
6	Annexin A2(ANXA2)	P07335	38/7.5	7	26	0.0150	6.0722 ± 5.5431
7	Heterogeneous nuclear ribonucleoproteins A2/B1(hnRNP A2/B1)*	P22626	37/9.0	16	43	0.0050	10.7775 ± 9.5547
8	Cathepsin D heavy chain(CTSD)	P07339	27/5.6	12	61	0.0012	4.3552 ± 3.7703
9	Protein kinase C and casein kinase substrate in neurons protein 1 (PACSIN1)	Q9BY11	51/5.1	5	24	0.0028	2.6026 ± 1.2147
10	Glutathione S- transferase P(GSTP)*	P09211	23/5.4	5	44	0.0026	3.8757 ± 2.2198
11	Peroxiredoxin-1(Prx-1)*	Q06830	22/8.3	8	59	0.0140	4.2744 ± 4.1568
12	Poly(rC)-binding protein1(PCBP1)*	Q15360	37/6.7	12	63	0.0070	2.7401 ± 0.5321
13	Aldo-keto reductase family 1member C3 (AKR1C3)	P42330	37/8.1	7	25	0.0040	2.7762 ± 1.1820
14	Glycineamidinotransferase (GATM)	P50440	44/6.4	8	23	0.0390	2.7839 ± 0.4680
15	Keratin, type II cytoskeletal 8 (Keratin 8)*	P05787	54/5.5	9	25	0.0020	3.5063 ± 0.6423
**Down-regulation proteins**							
16	Actin, cytoplasmic 1 (Actin 1)*	P60709	42/5.3	6	30	0.0160	0.2603 ± 0.1539
17	Heterogeneous nuclear ribonucleoproteins H3(hnRNPH3)	P31942	37/6.4	5	28	0.0015	0.6028 ± 0.6229
18	Peroxiredoxin-2(Prx-2)*	P32119	22/5.7	6	24	0.0250	0.2376 ± 0.1202
19	Carbonic anhydrase2(CA2) *	P00918	29/6.8	6	33	0.0130	0.3675 ± 0.1270
20	Alpha-enolase*(ENO1)	P06733	47/7.0	16	42	0.0030	0.2898 ± 0.1340
21	Alcohol dehydrogenase 1C (ADH1C)	P00326	40/8.6	7	49	0.0280	0.2287 ± 0.1680
22	Electron transfer flavoprotein subunit b (ETFB)	P38117	28/8.3	6	26	0.0240	0.2655 ± 0.1415
23	Voltage-dependent anion-selective channel (VDAC)*	P21796	31/8.6	7	39	0.0120	0.4474 ± 0.0297

a) Swiss-Prot accession no.

b) Theoretical value

c) Sequence coverage %

* The proteins have been found in gastric cancer previously.

### Immunohistochemical and western blot analysis for HSP 27, 60, and Prx-2 in GCA

**Figure 4:**
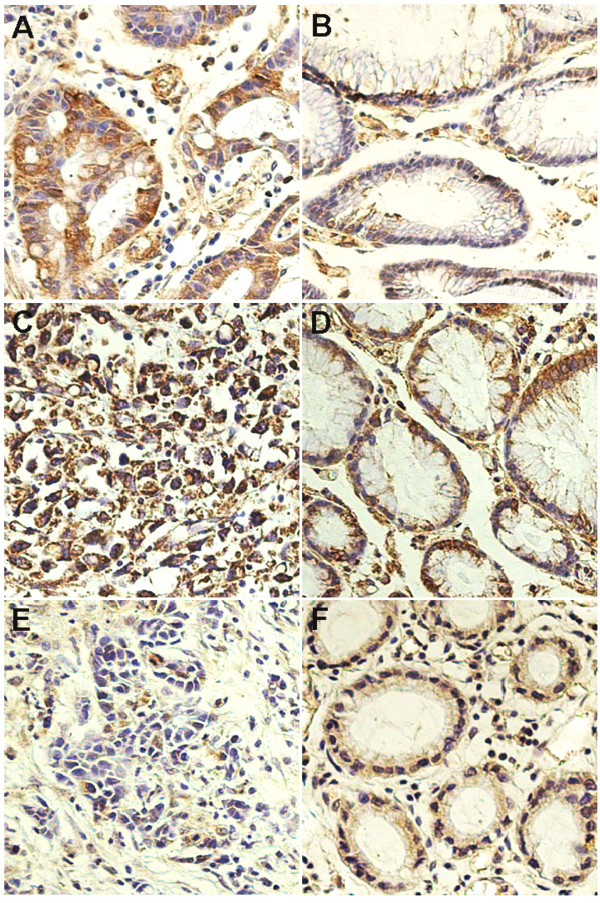
**Immunohistochemical analysis for HSP27, 60, and Prx-2 in human normal gastric cardia and GCA**. Expressions of HSP27 (A), 60(C), and Prx-2 (E) were in the cytoplasm of tumour cells; Expressions of HSP27 (B), 60(D) and Prx-2 (F) were found in cytoplasm of normal columnar epithelial cells; Magnifications: A-F × 40, counterstained with hematoxylin.

**Figure 5:**
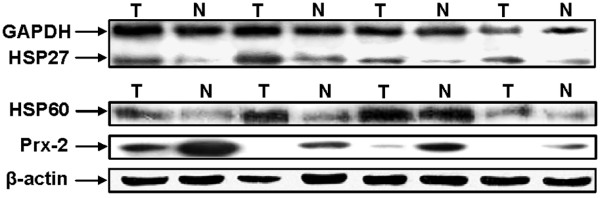
**Western blot analysis for validating the increased expression of HSP27, 60, and decreased expression of Prx-2 in GCA tissues**. GAPDH and β-actin were used as references.

To further investigate whether HSP27, 60, and Prx-2 are expressed in tissues and to determine which cells express these proteins, immunohistochemical analysis was performed using formalin-fixed and paraffin-embedded tissue specimens that were matched with 2-DE samples. The expression of HSP27 and 60 could be seen in all the cytoplasms of carcinoma cells, while it could be seen in some of the cytoplasms of columnar epithelial (Fig. 4A–D) in non-tumour tissues. In contrast, Prx-2 was almost not found to be expressed in the carcinoma cells but rather in some of the cytoplasms of columnar epithelial cells (Fig. 4E, F). To examine these protein expression levels, western blot analysis was also performed using the same tissues (Fig. 5). As expected, HSP27 and 60 were found to be consistently highly expressed in tumour tissue, whereas Prx-2 was suppressed.

## Discussion

Cardia is the anatomical borderland between the oesophagus and the stomach; hence, there is constant controversy concerning their relationship in terms of epidemiology, clinical features, and classification among esophageal adenocarcinoma (EA), GCA, and DGA. To compare their protein profiles, we searched the literature to find these proteins. Thirteen out of 23 proteins have been found in DGA previously (Table 2). Among them, HSP27, 60, 70, PDIA3, and CA2 have the same expression pattern in GCA and DGA. In contrast, a low-level expression of HSP27 was found in EA [16-20]. Therefore, the tumourigenesis of GCA is different from EA and DGA, and thus GCA should be a distinct pathological entity.

Cellular heterogeneity has been a significant barrier to the molecular analysis of normal and diseased tissues. First described by Emmert-Buck et al. in 1996, laser capture microdissection is a powerful and precise technique used to study protein changes in a particular subset of cells in a low-maintenance system that is easy to operate [8]. As usual, various histochemical stains of the tissue section are used to guide the dissection process. However, several cases have shown moderate to dramatic effects of staining on protein separation by 2-DE [21,22]. To eliminate staining as a potential detrimental effect, navigated LCM has been developed recently. It uses a stained section to guide the dissection of the unstained adjacent specimen [23]. This technique has been applied successfully in the study of brain samples [15]. In addition, due to the typical morphological characteristics of the gastric cardiac superficial columnar cell and the cardiac gland, they are easy to identify on a dehydrated section without staining. Therefore, navigated LCM became an optical strategy in our proteomics approach. In this research, the numbers of LCM-derived nonmalignant and malignant cells varied substantially and were often small compared with the whole undissected cryostat tissues, notwithstanding the profiles that were very similar between the LCM samples and the undissected ones. This indicated that the navigated LCM may be a feasible approach for the study of gastric cardiac tissue and that it is compatible with 2-DE and MALDI-TOF MS.

We obtained the protein profile of GCA by comparing the changes in the protein profiles of surrounding non-tumour tissues. To reduce individual differences, tissue samples were obtained from the same patient, enabling us to study differential protein expression under similar genomic background. To the best of our knowledge, we reported the first proteomic analysis of GCA. Of the 27 selected protein spots, 23 proteins in the molecular mass range of 15 to 75 kDa and with an isoelectric point between 3 and 10 were identified by 2-DE and MALDI-TOF MS. Most of these proteins were described previously as differentially expressed either at the mRNA level or at the protein level in other types of human cancer.

HSPs are a group of stress proteins induced by various types of environmental and pathophysiologic insults. In this study, co-up-regulations of HSP27, 70, and 60 were found in the gastric cardiac tumour tissues. As chaperones, HSP27 and HSP70 could inhibit apoptosis by interacting with apoptosome and the caspase of activation complex [24], underlying their roles in tumour progression and resistance to treatment [25]. Numerous studies indicate that HSP27 and HSP70 overexpressions signal a poor prognosis and predict a poor response to chemotherapy. HSP60 appears to be a key endogenous inflammatory mediator and is presumably released by damaged cells. Moreover, together with HSP70, HSP60 has a role in antigen presentation in malignant diseases [26]. HSPs are also overexpressed in breast, colorectal, gastric, and prostate carcinomas [10,25]. Therefore, overexpression of HSP27, 70, and 60 may be useful biomarkers for carcinogenesis in GCA and may be associated with the degree of differentiation and the prognosis of GCA.

Among identified proteins, two proteins are involved in the regulation of transcription and expression of tumour-associated genes. PCBP1, up-regulated in GCA, is an RNA chaperone post-transcriptional regulator. It has been demonstrated that PCBP1, together with PTB-1 and hnRNPK, controls some proto-oncogene genes and apoptosis-related genes expression, such as Bag-1, *c-*myc, Apaf-1, XIAP, and DAP5, through stimulating the activity of internal ribosome entry segment (IRES). PCBP1 is required to open the RNA in the region containing the ribosome entry window, while PTB-1 may be required for ribosome recruitment [27,28]. Recently, Pickering, B.M. et al. described that the glycan parts of PCBP1 might be related to metastatic ability and might play a role in hepatocellular carcinoma metastasis [27]. Then the up-regulation of PCBP1 may regulate the cap-independent mechanism of translation initiation of cardiac tumour-associated genes in the development of GCA. As a tumour suppressor, Alpha-enolase can regulate the c-myc promoter activity in the form of a c-myc binding protein (MBP-1). MBP-1 can then bind to the P2 element in the c-myc promoter and compete with the TATA-box binding protein (TBP) to suppress the transcription of c-myc [29,30]. On the other hand, down-regulation of Alpha-enolase is in accordance with the work of Chang YS et al. where it was found that Alpha-enolase predicted aggressive biological behaviour and is associated with poor survival in non-small cell lung cancer [29].

It is noteworthy that three antioxidant enzymes, namely, Prx-1, Prx-2, and GSTP, were identified in gastric cardiac tumour. All are involved in the removal of reactive oxygen species (ROS) which can induce cellular senescence and apoptosis and therefore function as antitumourigenic species [31]. Thus, we presumed that overexpression of Prx-1 and GSTP may protect gastric cardiac tumour cells from apoptosis by scavenging ROS in these cells. Moreover, evidence suggests that enhanced antioxidant mechanisms in tumour cells in vivo contribute to chemoresistance and poor prognosis. Increased expressions of Prx-1 and GSTP have been detected in hepatocellular [32] and pancreatic carcinoma [33]. The down-regulation of Prx-2 in GCA may therefore imply its role as a tumour suppressor, which was controversial in some other studies [34]. However, Furuta, J. et al. recently found that Prx-2 was silenced in melanomas due to aberrant methylation of 33 CpG islands [35]. Therefore, the disregulations of antioxidant enzymes in GCA may represent tumour cells with a microenvironment which is advantageous to their survival and proliferation.

The other up-regulated proteins involved in physiological processes are ANXA2, ANXA4, and hnRNPA2/B1. Annexins are Ca^2+ ^and phospholipid binding proteins that act as regulators of membrane fusion and possess the structural properties necessary to form ion channels [36]. While ANXA2 and ANXA4 are associated with several physiological processes (e.g., signal transduction, cellular differentiation, and proliferation), their roles in GCA tumourgenesis have not been described previously. ANXA2 was overexpressed in advanced gastric carcinomas and could have contributed to its progression [37]. In relation to this, Zimmermann et al. reported that ANXA4 plays an important role in the morphological diversification and dissemination of renal cell carcinoma [38]. Therefore, the overexpression of ANXA2 and ANXA4 may be related to the malignant transformation of gastric cardia epithelia. hnRNPA2/B1 is a member of a large family of hnRNP proteins involved in various functions including the regulation of transcription, mRNA metabolism, and translation [39]. He, Y. et al. presented evidence that hnRNP A2/B1 may play an important role in cell proliferation through the regulation of BRCA1 and p21 expressions [40]. Guo, W. et al. found that p21 might have an effect on GCA development [41]. Moreover, hnRNP A2/B1 is a target antigen for MG7, which is an early gastrointestinal cancer-specific monoclonal antibody [42]. Thus, further studies are needed to discover the pathogenesis of hnRNP A2/B1 in GCA.

CAs are physiologically important enzymes that catalyse reversible conversions of carbon dioxide to bicarbonate [43]. There are at least 13 active isoenzymes that have been identified in mammals. They are involved in many biological processes such as pH homeostasis and ion transport. CA2 is a very efficient enzyme and is expressed in most organs of the alimentary tract. It has high expression in the gastric and intestinal epithelia [44,45]. Its main physiological functions are to regulate the acidity of gastric juice, assist in forming a HCO3^-^, cover the epithelium, and protect it from digestion [44]. A significantly low expression of CA2 may make it difficult for CA2 to maintain its function for normal gastric cardiac cell growth and could therefore lead to the progression of malignant transformations. Recently, both CA1 and CA2 were reported to have down-regulations in colorectal and gastric tumours and were considered to participate in cancer biological aggressiveness and synchronous distant metastasis [46,16].

ADH1C belongs to the zinc-containing alcohol dehydrogenase family. It takes part in alcohol metabolic process and can catalyse the oxidation of approximately 80% of ethanol to acetaldehyde, a known toxic and carcinogenic compound [47]. Increased acetaldehyde production has been implicated in the pathogenesis of colorectal, breast, and hepatocellular cancers [48-50], thereby indicating that a high intake of alcohol is associated with tumourgenesis. However, our study's results initially found that ADH1C was significantly reduced in GCA. In addition, the role of alcohol consumption in GCA is controversial. Some studies considered it as a risk factor for GCA, but a case control study in Sweden revealed that there was no positive correlation between alcohol consumption and esophageal or cardiac adenocarcinoma. Therefore, further studies are required to elucidate the molecular mechanism of ADH1C in the development of GCA.

## Conclusion

We applied navigated LCM to the proteomic study of GCA and compared the differential expressions of protein in GCA tissues and surrounding non-tumour tissues. The current study is therefore the first report on proteome analysis in GCA. Twenty-three proteins were shown to have significant differential expressions in GCA tissues compared with surrounding non-tumour tissues. Most of the proteins identified were not reported in GCA. These results may help elucidate the molecular mechanisms of GCA carcinogenesis and could provide potential clinical biomarkers for early detection and identification of therapeutic targets. However, further functional analysis is necessary to elaborate the roles of these cancer-associated proteins.

## Competing interests

'The author(s) declare that they have no competing interests.

## Authors' contributions

YC carried out the LCM, 2-DE, and MALDI-TOF MS studies, and drafted the manuscript. YL carried out the immunohistochemical and western-blot, as well as the statistical analysis. JG and YW participated in the design of the study. JZ conceived the study, participated in its design and coordination, and also helped in drafting the manuscript. All authors read and approved the final manuscript.

## Pre-publication history

The pre-publication history for this paper can be accessed here:


